# A computational model of the competitive effects of ESG

**DOI:** 10.1371/journal.pone.0284237

**Published:** 2023-07-21

**Authors:** Evangelos Katsamakas, J. Manuel Sanchez-Cartas

**Affiliations:** 1 Fordham University, New York, NY, United States of America; 2 Universidad Complutense de Madrid, Campus de Somosaguas, Madrid, Spain; University of Almeria: Universidad de Almeria, SPAIN

## Abstract

Environmental and social initiatives within firms, commonly grouped under the ESG term, have attracted significant business interest. However, the mechanism that links ESG investment to firm performance is unclear. We develop a computational model that helps clarify the competitive effects of ESG. In our model, ESG investment attracts consumers, but it can have additional effects on companies, such as reducing production costs, increasing product value, or both. Computational experiments show that ESG intensifies competition when it has such additional effects in addition to attracting consumers. However, ESG can lead to a winner-take-all dynamic in which a firm with an initial advantage dominates the market. Moreover, firms can use strategic disclosure of information to reduce their ESG investments, softening competition. This research contributes to the ESG literature by explaining the strategic impact of firms’ ESG investments and the conditions under which firms can do well by doing good in a competitive setting.

## Introduction

Environmental, Social, and Governance (ESG) and related Corporate Social Responsibility (CSR) issues have attracted significant interest in business [1–3]. Firms implement social and environmental sustainability initiatives to integrate purpose and profit [4–6], manage risk, attract and retain customers and employees, and gain a competitive advantage [4–6]. However, what is the link between ESG and firm performance, and can a firm do well by doing good? These questions have long attracted the attention of scholars [7–13], but many aspects of ESG need to be clarified [14, 15], and empirical research finds conflicting hypotheses and mixed results [16–19].

While the mixed empirical results call for more empirical research, they also call for more modeling research to clarify the mechanisms that connect ESG and firm performance. In that direction, we must first consider effects that are characteristic of ESG. One main effect is that ESG helps attract consumers, an observation supported by several research studies [20–24] and industry surveys showing that consumers prefer companies acting responsibly on social and environmental issues [3, 25–30]. However, attracting consumers is not the only effect associated with ESG investment. Lowering costs is another potential effect of investing in ESG [31]. For instance, a firm may use smart meters and sensors (IoT) to lower energy costs or use data and analytics to make business processes more efficient. More generally, digital technologies can help lower operation costs toward achieving the firm’s ESG goalsfor example, using big data analytics in CSR helps improve CSR performance [32, 33]. Alternatively, by investing in ESG, firms can attract and retain innovative talent to lower the firm’s marginal cost. Additionally, ESG investment in product innovation can increase the value of a firm’s products and the consumers’ willingness to pay [22, 23, 34]. It is also likely that ESG could increase costs, as the production of green products requires specific costly techniques. Therefore, ESG attracts consumers, but it also has multiple other direct effects on a firm depending on the ESG investment.

This article provides an integrated framework to study and compare multiple effects of ESG. We build an agent-based computational model that clarifies the mechanisms through which ESG impacts firms in a competitive setting. We consider the main effect of ESG, attracting consumers, along with other effects such as lower production costs or increased product value. Computational experiments allow us to see how various ESG direct effects interact with the market structure to determine the competitive outcomes of ESG investment. The main research question we answer is: How does ESG investment impact firms in a competitive setting? Answering this question can help shed some light on whether and when firms can do well by doing good. While ESG is a most recent term, this article treats ESG and CSR as interchangeable, following [16] that discusses a firm’s ESG/CSR and social responsibility profile. That profile characterizes how a firm manages risks and opportunities associated with social, environmental, and governance dimensions.

A few papers develop game-theoretic or simulation models related to ESG/CSR, as we do in this research. They study CSR assuming that CSR-compliant operations are more costly than the alternative [35], propose a game-theoretic auction model of integrating sustainability into the business model [36], and consider the competition of two firms with different marginal costs [37, 38]. In this theoretical literature, a specific ESG feature is chosen and evaluated in a theoretical framework. However, we lack a study that considers multiple ESG effects, and their interactions, in a common framework for comparison. Moreover, no other ESG study has considered the concept of attracting consumers despite being a crucial feature of ESG. We fill the latter two research gaps.

Our research finds that ESG does not affect competition when ESG investment attracts consumers only. However, when ESG investment is used for process or product innovation, in addition to attracting consumers, it intensifies competition, lowers prices and profits, and increases consumer welfare. We also find that ESG can generate winner-takes-all dynamics; therefore, it is in a firm’s interest to invest in ESG early, getting ahead of its competitors that may exit the market if they lag in ESG investment. However, if all firms invest in ESG, profits are dissipated. We also find that firms have the incentive to signal their ESG investment to avoid excessive investments and soften competition.

This article makes several contributions to the literature. It contributes an integrated framework to evaluate and compare multiple effects of ESG. It also contributes by modeling a novel effect of ESG investment: firms invest in ESG to attract consumers. This is a significant modeling innovation as consumers move to the company rather than the company moving to the consumers; traditionally, the modeling focus has been the optimal positioning of firms. Our work helps clarify the mechanism linking a firm’s ESG initiatives and performance and provides new lessons for managers to resolve the overarching puzzle of whether a firm can do well by doing good.

## Methods

The results of this research are obtained through computational experiments. The details of the agent-based computational model are presented next. Other researchers have used agent-based computational modeling to study technological innovation, market dynamics, and other economic problems [39–44].

### Model

We develop an agent-based computational model to study the impact of ESG investment in a competitive setting.

### Baseline model

Our model is based on the classic Hotelling model, which has been used widely in the study of market competition [45–48]. The model assumes two types of agents: Consumers and firms. Two horizontally differentiated firms are at locations 0 and 1 on a line with unit length. Consumers are uniformly distributed along the line and demand one unit of product. A consumer compares two products and chooses the one that gives him/her the highest utility. Consumer utility for a product depends on its intrinsic value, price, and the mismatch between the firm’s and the consumer’s locations. This mismatch represents the difference between the consumers’ tastes and the firm’s offering. Formally, the utility for the consumer-*j* located at *x* from purchasing firms-*i*’ product located at *l*_*i*_ ∈ [0,1] is

$$u_{j}^{i}=v-p_{i}-t\left|x_{j}-l_{i}\right|$$ (1)

The parameter *v* captures the intrinsic value of the product that we assume is high enough (3/2t< v) to guarantee that all consumers participate. *p*_*i*_ represents the price set by firm *i*. Parameter *t* captures the level of horizontal differentiation (mismatch) between the competing firms—a smaller value of *t* implies a lower level of differentiation and higher competition intensity. The mismatch cost *t*|*x*_*j*_ ‒ *l*_*i*_| captures the consumer heterogeneity. In this symmetric framework, firm-i demand is as follows:

$$n_{i}=\frac{1}{2}+\frac{1}{2}\frac{\left(p_{j}-p_{i}\right)}{\text{t}}$$ (2)

Each firm faces this demand and sets prices to maximize profits (*max*_*pi*_ *π*_*i*_ = (*p*_*i*_ ‒ *c*_*i*_)*n*_*i*_) where *c*_*i*_ is the marginal cost. In this situation, firms set prices simultaneously p* = *c* + *t*, and in equilibrium, the market is equally shared.

### Main model: Firms invest in ESG to attract consumers

**Fig 1:**
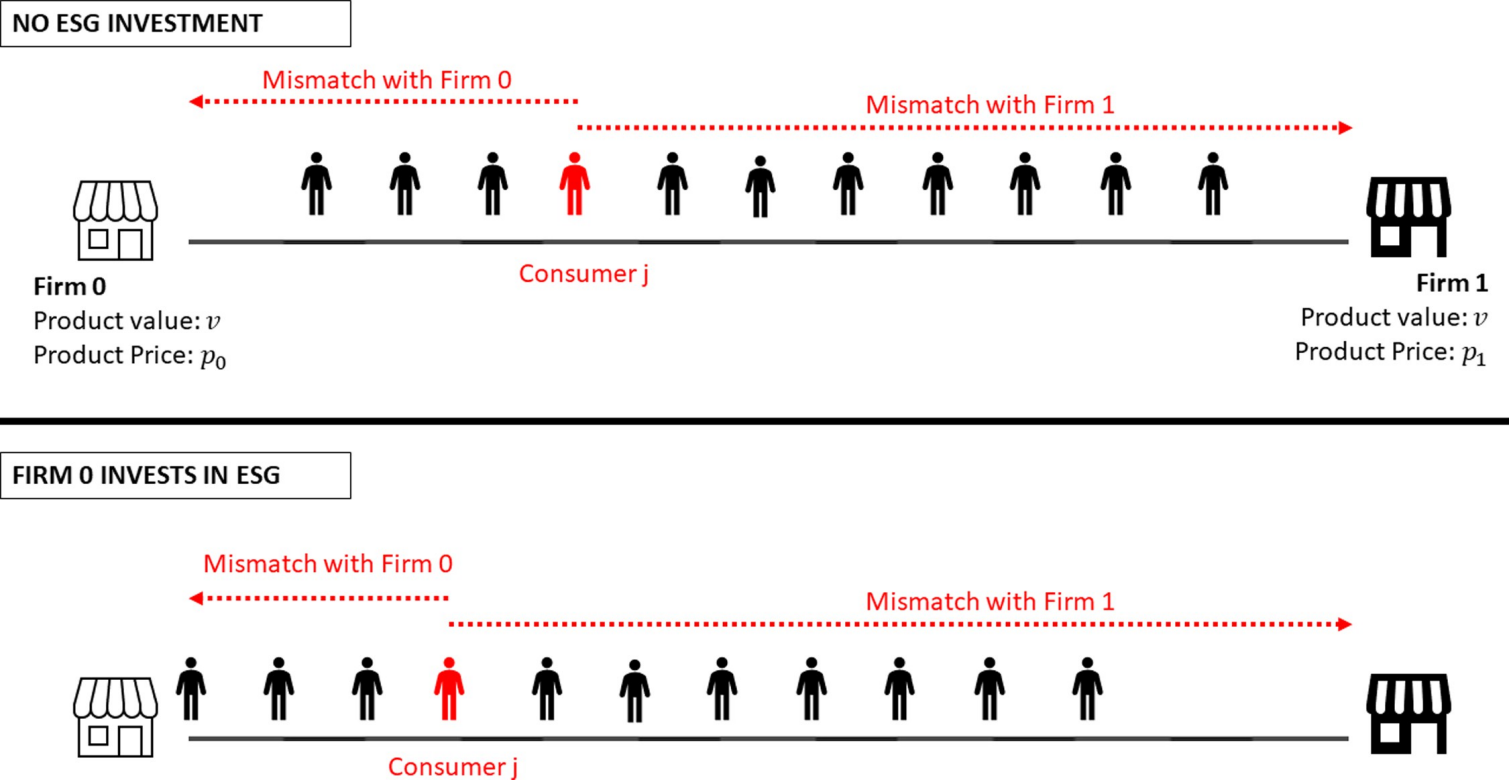
Graphical comparison of the baseline model (no ESG investment) and the case that firm 0 invests in ESG to attract consumers.

We add a novel modeling feature to the baseline model: firms can invest in ESG to educate consumers, modifying how consumers perceive their brands. A firm’s ESG investment attracts consumers to the firm and changes the distribution of consumers, as in the case of persuasive advertising [49]. Formally, after the company has invested ϵ in ESG, the distance from consumers to that company will be *max*{|*x*_*j*_ ‒ *l*_*i*_| ‒ ϵ, 0}. Fig 1 illustrates the effect of ESG investment.

Because investing in ESG is costly, the profit of each company is:

$$\pi_{i}=\left(p_{i}-c_{i}\right)n_{i}-\delta \epsilon$$ (3)

where ϵ is the ESG investment, and δ is the marginal cost of each additional unit of ESG investment. We converge to the baseline model if δ = 0. In contrast with the baseline model, firms set prices and the ESG level (*p*,ϵ) that maximize their profits (instead of only prices).

Consumers choose the firm that maximizes their utility. However, the demand functions differ from the demand functions of the baseline model shown in Eq. (2) because firms modify the mismatch cost (|*x*_*j*_ ‒ *l*_*i*_| ‒ ϵ) of consumers according to the level of ESG investment ϵ. The new demands must be computed.

To compute the demands, prices, and ESG levels, we use the algorithm proposed in [50]. The algorithm simulates a tâtonnement process, where firms may increase, keep, or decrease their prices at each iteration by considering how those price changes affect consumer utilities (and demands).

### Summary of scenarios

In the first scenario, firms invest in ESG to attract consumers. In the other four scenarios, besides attracting consumers, ESG investment has additional direct effects: reduction of marginal cost, increase of product value, reduction of marginal cost and increase of product value, increase of marginal cost and product value. This analysis takes into account process innovation that lowers the marginal cost (*c*_*i*_) and product innovation that increases the product value (*v*) [51–53]. All scenarios consider the ESG investment levels, prices, profits, and welfare.

## Results

Next, we present the results for each scenario.

### ESG investment that attracts consumers only

In our first scenario, firms invest in ESG to attract consumers. By attracting consumers, a firm reduces the consumer mismatch cost allowing the firm to extract a higher surplus. At the same time, by attracting consumers, a firm reduces the competitor’s influence. However, attracting consumers can be costly. Marketing practitioners know that changing a brand image requires time and money; in the short run, price competition can be a better way to attract consumers than investing in ESG. Therefore, firms face a non-trivial trade-off.

We find that the equilibrium coincides with the equilibrium of the baseline model. If the investment is costly and does not radically modify consumer perceptions, a marginal price change helps the firm gain more consumers than a small change in ESG investment. Therefore, firms prefer to compete in prices only. We also ran a sensitivity analysis assuming that ESG can modify consumer perceptions to varying degrees (3%, 6%, 9%, and 12%), shown in S1 Appendix in S1 File. The result holds for all our cases as long as we assume that ESG is costly. This result shows that when ESG does not precede process or product innovations, it may not be optimal to invest in it. In other words, although attracting consumers and employees is a common feature of ESG investments, this effect alone does not justify investing in ESG. If companies only want to gain market share, other alternatives to ESG may work just as well, such as sales or price reductions.

### ESG investment that reduces costs

In some cases, firms may invest in ESG to reduce costs and attract consumers. As already mentioned, digital technologies like IoT, data, and artificial intelligence (AI), can help reduce energy consumption and lower the costs of operation or production. Alternatively, by investing in ESG, firms can attract and retain innovative talent to lower the firm’s marginal cost. In this scenario, ESG investment plays a dual role, modifying consumer perceptions and reducing marginal costs.

**Fig 2:**
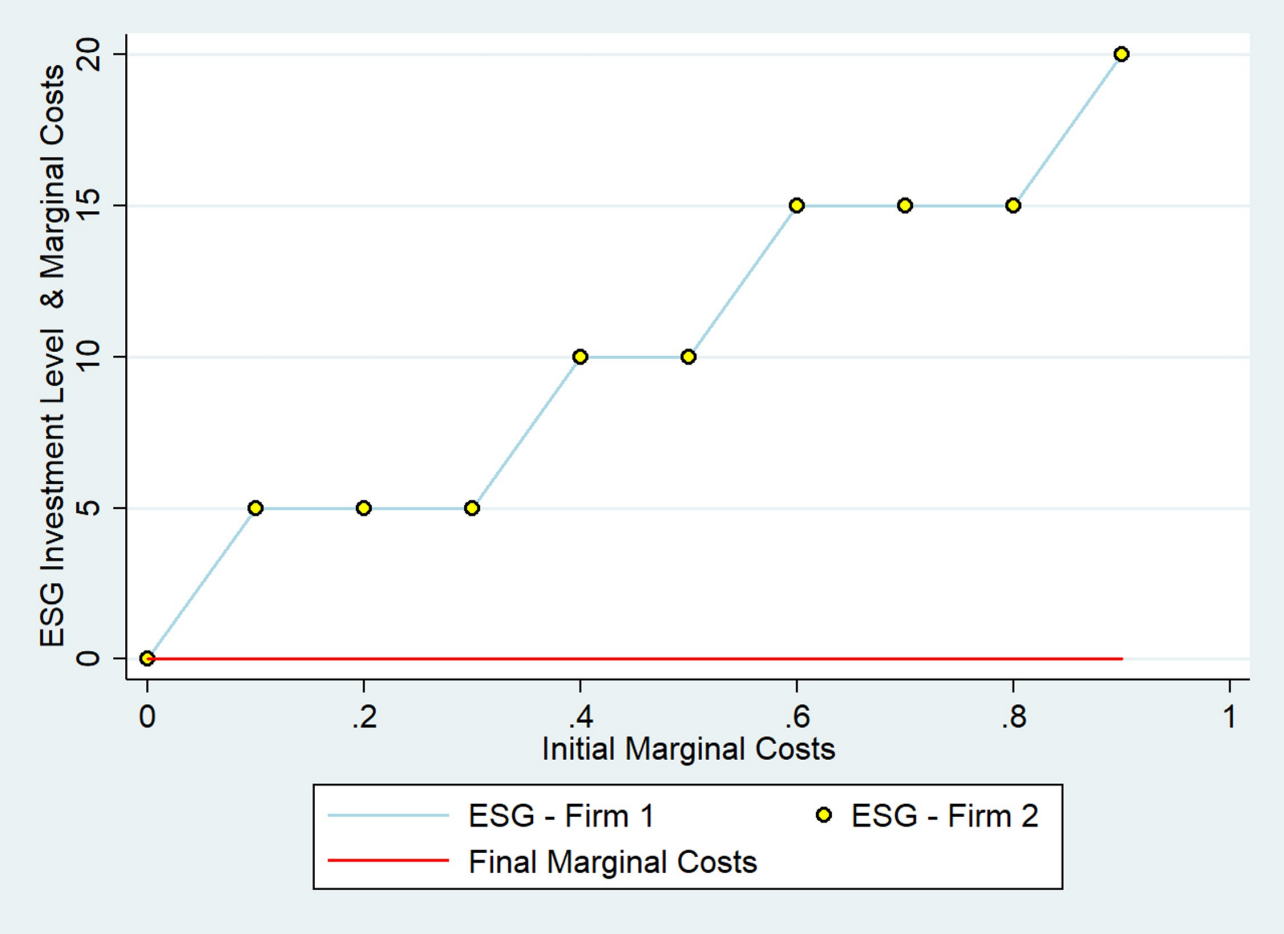
Effects of ESG on marginal costs.

We find that firms have an extra incentive to invest in ESG because it triggers a reduction of their marginal costs. The higher the initial marginal costs, the greater the incentive to invest in ESG (Fig 2). In all cases, firms invest enough to reduce their marginal costs to zero independently of the initial marginal costs. However, given the symmetric framework, the distribution of consumers does not change, but profits dissipate. The basic intuition of this result is similar to the one in the [54] model, in which two ex-ante identical firms can invest in a cost-reducing technology. In that model, early adoption prevents adoption by the competitor, and that incentive leads to an equilibrium in which any first-mover advantage dissipates. Here, we have a similar result with the difference that ESG influences the allocation of consumers, which makes the investment more profitable a priori. However, given the symmetric framework, no change in user distribution occurs in equilibrium. This result is also robust to different levels of differentiation. In all cases tested, the intuition remains the same.

### ESG investment that increases product value

In this scenario, ESG investment attracts consumers and increases product value. For example, green products have become trendy despite higher prices because they promise a lower ecological impact. The increase in intrinsic product value can be seen as a quality increase that raises the willingness to pay. However, the difference between an increase in quality and ESG investment is that the latter may also affect consumer tastes. In this sense, we consider that ESG influences the consumer perception of the brand and the intrinsic value of a firm’s offering.

In this scenario, we have two variables that significantly alter the equilibrium level of ESG: mismatch costs (differentiation) and the starting price.

**Fig 3:**
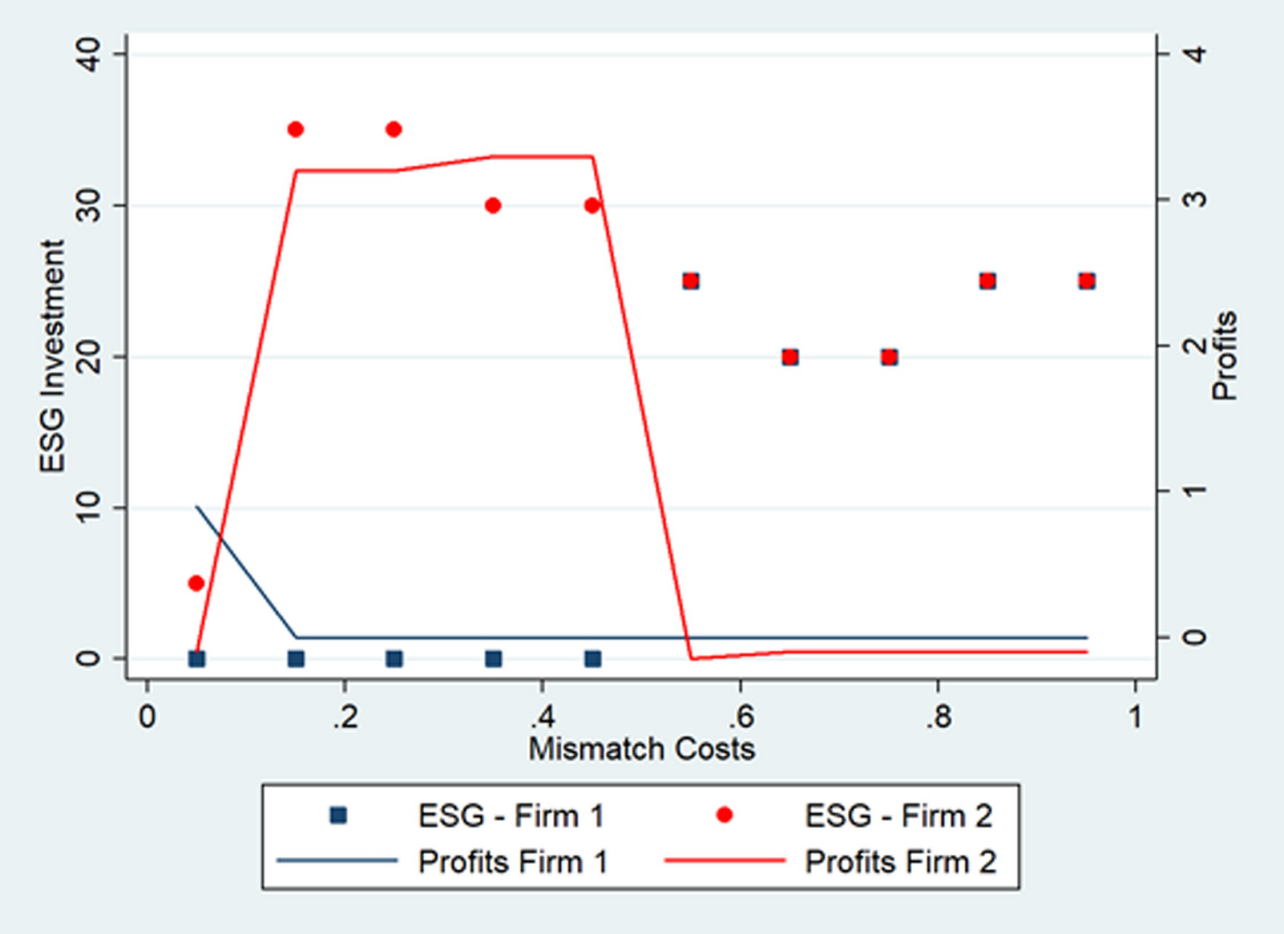
ESG investment and profits when ESG increases the intrinsic product value.

When mismatch costs (differentiation) are low (below 0.5), one firm can keep the whole market and earn significant profits by expelling the competitor (Fig 3). The winning firm outperforms the competitor by investing more than her. However, keeping competitors out of the market becomes impossible as differentiation increases (mismatch costs above 0.5). One firm remains ESG leader and the other a follower, but both dissipate profits and invest the same. Those effects seem robust to the presence of the reallocating effect of ESG and suggest the double-edged nature of ESG: ESG can increase the value created, but it creates a leader-follower dynamic that harms both companies.

**Fig 4:**
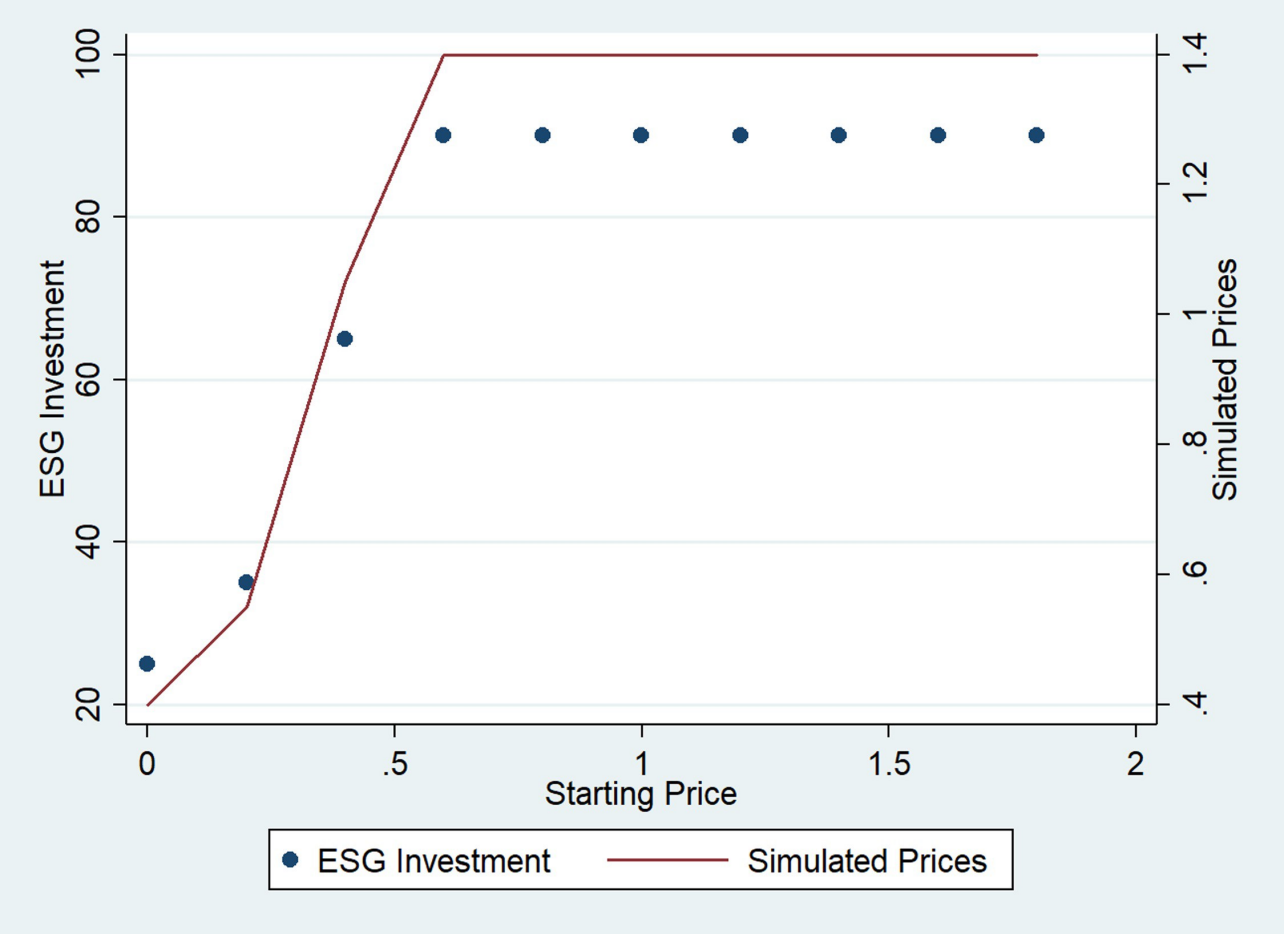
Prices and ESG investment. Different starting prices.

Another interesting finding is how the initial price affects the final ESG investment (Fig 4). If starting prices are too low, firms cannot invest and remain stuck in a suboptimal solution. However, firms can converge to a stable symmetric interior equilibrium as the initial price increases (starting prices above 0.5 in Fig 4). Profits are dissipated, in contrast with previous economic theory [55], because the reallocation effect of ESG creates an extra incentive to invest. This result suggests that not investing in ESG can be an equilibrium in industries with fierce competition. In other words, ESG in already competitive markets might not be desirable from the firms’ point of view. If prices are low, firms do not invest, which suggests that firms might alternate between price and non-price competition.

### ESG investment that reduces marginal costs and increases product value

In this scenario, ESG investment attracts consumers and, at the same time, reduces marginal cost and increases product value. Do these two effects reinforce each other, or do they present decreasing marginal returns?

We find that competition erodes profits similar to previous scenarios and, although in general prices are higher, welfare is higher too because of the increase in intrinsic product value.

**Fig 5:**
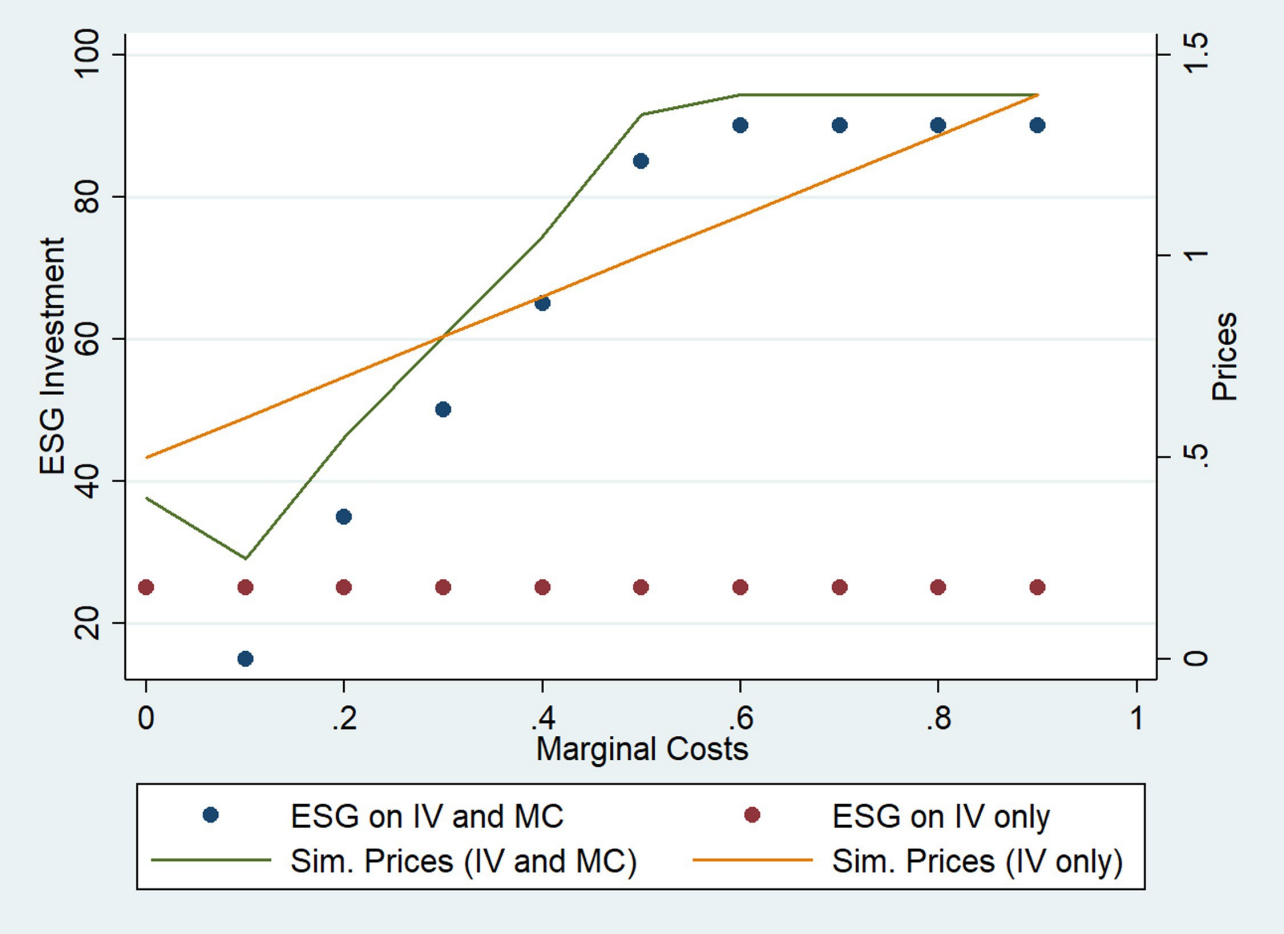
Comparison of effects of ESG on marginal costs (MC) and intrinsic product value (IV).

However, we find a nonlinear behavior of prices and ESG investments that differs from the linear behavior observed when only one dimension is considered (Fig 5). Therefore, when ESG affects multiple dimensions simultaneously (consumer perceptions, costs, product value), we should refrain from studying the individual effects of ESG. A testable hypothesis about the impact of ESG in different industries follows: if ESG increases intrinsic product value (IV) and reduces costs (MC), a nonlinear ESG behavior is likely (green line in Fig 5), whereas if ESG reduces costs only, a more linear impact is likely (orange line in Fig 5).

### ESG investment that increases product value and marginal costs

**Fig 6:**
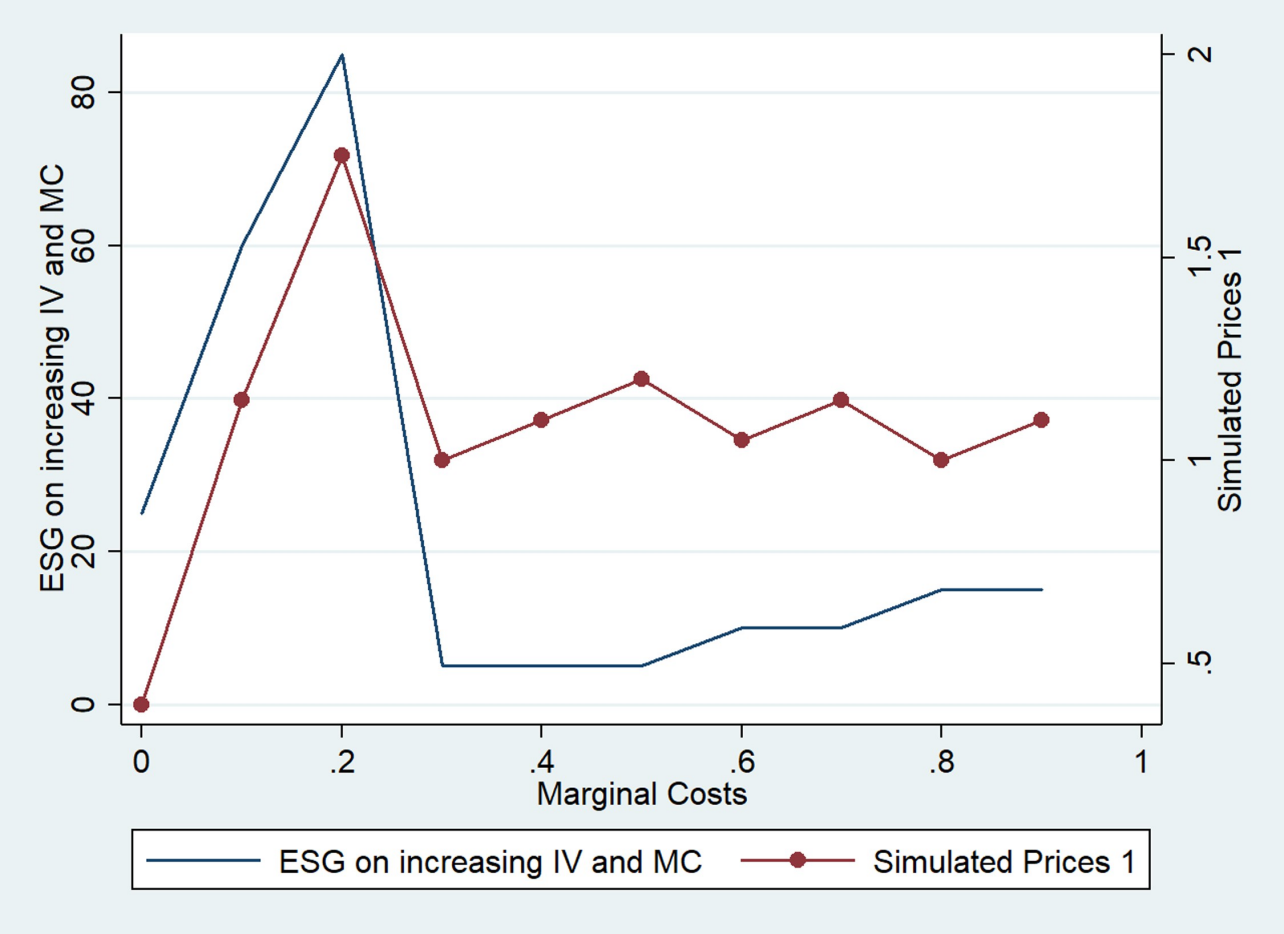
ESG effects: Increasing marginal cost and intrinsic value.

Some ESG practices may have the combined effect of higher marginal costs and intrinsic product value. This scenario captures situations where ESG practices make production or operations more costly; for instance, a farm producing organic products incurs a higher marginal cost due to certifications and lower output. Fig 6 shows our results.

When marginal costs are low (smaller than 0.3 in Fig 6), firms invest heavily in ESG because they can profit quickly from the increased intrinsic product value, but both firms dissipate profits. However, as initial marginal costs increase, the incentive to invest weakens. The incentive to invest is the weakest for intermediate values of marginal costs, between 0.3 and 0.5. Nevertheless, the incentive becomes stronger as initial marginal costs keep increasing. Both firms have high marginal costs but can gain a competitive advantage by investing to increase their intrinsic product value. Thus, they keep investing. This time, the trade-off between marginal cost and ESG favors the gains from ESG more moderately.

These results suggest that ESG may not be a one-size-fits-all solution for all companies, and we should expect significant differences depending on the cost structures of each industry. Firms with low marginal costs, such as digital firms, may be more inclined to invest in ESG.

### Comparison of all scenarios

Table 1 depicts the prices, profits, ESG investment, and welfare of a representative market in all scenarios. ESG does not impact competition in the first scenario (ESG investment attracts consumers only). However, all other scenarios lead to lower prices and higher welfare. In other words, ESG creates a pro-competitive effect that benefits consumers, especially when ESG reduces costs and increases the intrinsic product value. Intuitively, there is more pressure to compete when both effects are combined, but the increase in the intrinsic value also creates the opportunity to set higher prices. That is why we observe a larger welfare than in previous scenarios but a similar price level.

**Table 1:** Comparison of ESG investment scenarios (marginal and mismatch costs equal to 1).

Scenario	Prices	Profits	ESG Investment	Consumer Welfare
Baseline model (no ESG investment)	1.85	0.95	0	263.45
ESG investment that only attracts consumers	1.85	0.95	0	263.45
ESG investment that attracts consumers AND reduces marginal costs (Fig 2)	1	0.6	20	530
ESG investment that attracts consumers AND increases product value (Fig 3)	1.3	0	25	1220
ESG investment that attracts consumers AND reduces marginal costs & increases product value (Fig 5)	1.4	0	90	3230
ESG investment that attracts consumers AND increases marginal costs & increases product value (Fig 6)	1.1	0.65	15	970

Due to increased competition, ESG investment reduces profits in all scenarios. This result shows a trade-off between purpose and profits for companies. Individually, they have incentives to invest in ESG to attract consumers and potentially benefit from process or product innovation. However, increased competition erodes profits when all firms do the same and invest in ESG. This situation raises new challenges for managers who seek to integrate purpose and profits, meeting the expectations of multiple stakeholders. In the next section, we discuss what else companies can do about those challenges.

### Extensions

We present results for two extensions. In the first extension, we study strategic commitment effects (two-stage game). In the second extension, we discuss asymmetric competition settings.

### Strategic commitment effects

It is common to find companies announcing their ESG investment or ESG projects [1]. For example, Google publicly announces its projects and the progress of its ESG investments [56]. By announcing their actions, companies are signaling their ESG efforts to their competitors. This situation can be represented by a two-stage game in which, in the first stage, firms set their investment levels and, in the second stage, compete on price.

In contrast with the simultaneous move game of the previous section, we find that firms do not dissipate profits, and in general, ESG investment and welfare are lower than before (Table 2). These results support the idea that there is a negative strategic commitment effect, and firms play a *puppy dog* strategy [57], whereby companies reduce their ESG investment to avoid appearing fierce to their competitors and thereby mitigate competition.

**Table 2:** Comparison of ESG investment scenarios. Marginal and Transportation costs at 1. Two-Stage Game.

Scenarios	Prices	Profits	ESG Investment	Welfare
Baseline model (no ESG investment)	1.9	1	0	248
ESG investment that only attracts consumers	1.9	1	0	248
ESG investment that attracts consumers AND reduces marginal costs	1.65	1	5	327
ESG investment that attracts consumers AND increases product value	1.9	1	15	718
ESG investment that attracts consumers AND reduces marginal costs & increases product value	1	1	25	1315
ESG investment that attracts consumers AND increases marginal costs & increases product value	1.15	1	15	954

Table 2 shows higher prices and profits and lower welfare than Table 1 in all cases. The main reason is that firms avoid overinvesting in ESG. Therefore, firms can use strategic disclosure of information to signal their ESG investment and avoid excessive investments.

**Fig 7:**
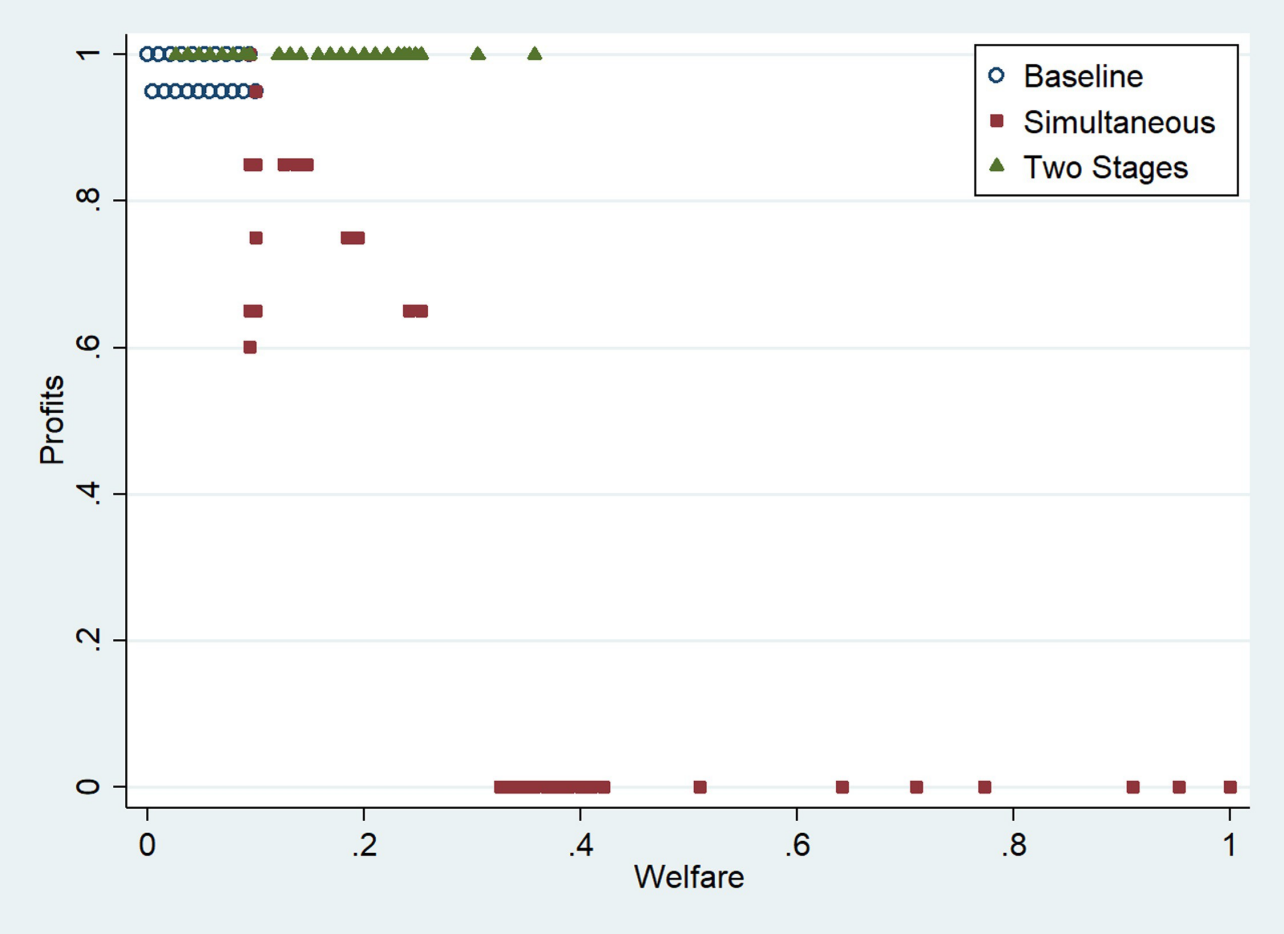
Normalized profits and welfare levels by type of game.

Fig 7 helps us illustrate this finding. Every point in Fig 7 represents one scenario under a specific combination of parameters (instead of one representative market as in Tables 1 and 2). To facilitate comparisons, we cluster the cases according to how firms make the ESG investment decision (simultaneous or two-stage). Since the scale of profits and welfare differs, we normalize the data to depict all the cases between 0 and 1. Fig 7 illustrates trade-offs between purpose and profits. Although it is possible to increase profits and welfare above baseline levels, there is a clear negative relationship between them. However, the interesting point about this negative relationship is the essential role that the competition assumptions (simultaneous or two-stage) play. Shedding light on the mixed results found in the empirical ESG literature, our research shows that it matters how firms compete and the type of ESG investment. Even the same type of investment can have opposite effects on firm performance depending on whether firms in that market signal their investment.

### Asymmetric competition

In this scenario, only one firm considers investing in ESG. We find that the firm that invests in ESG can attract all the consumers to its position, which alters how both firms compete in equilibrium.

**Fig 8:**
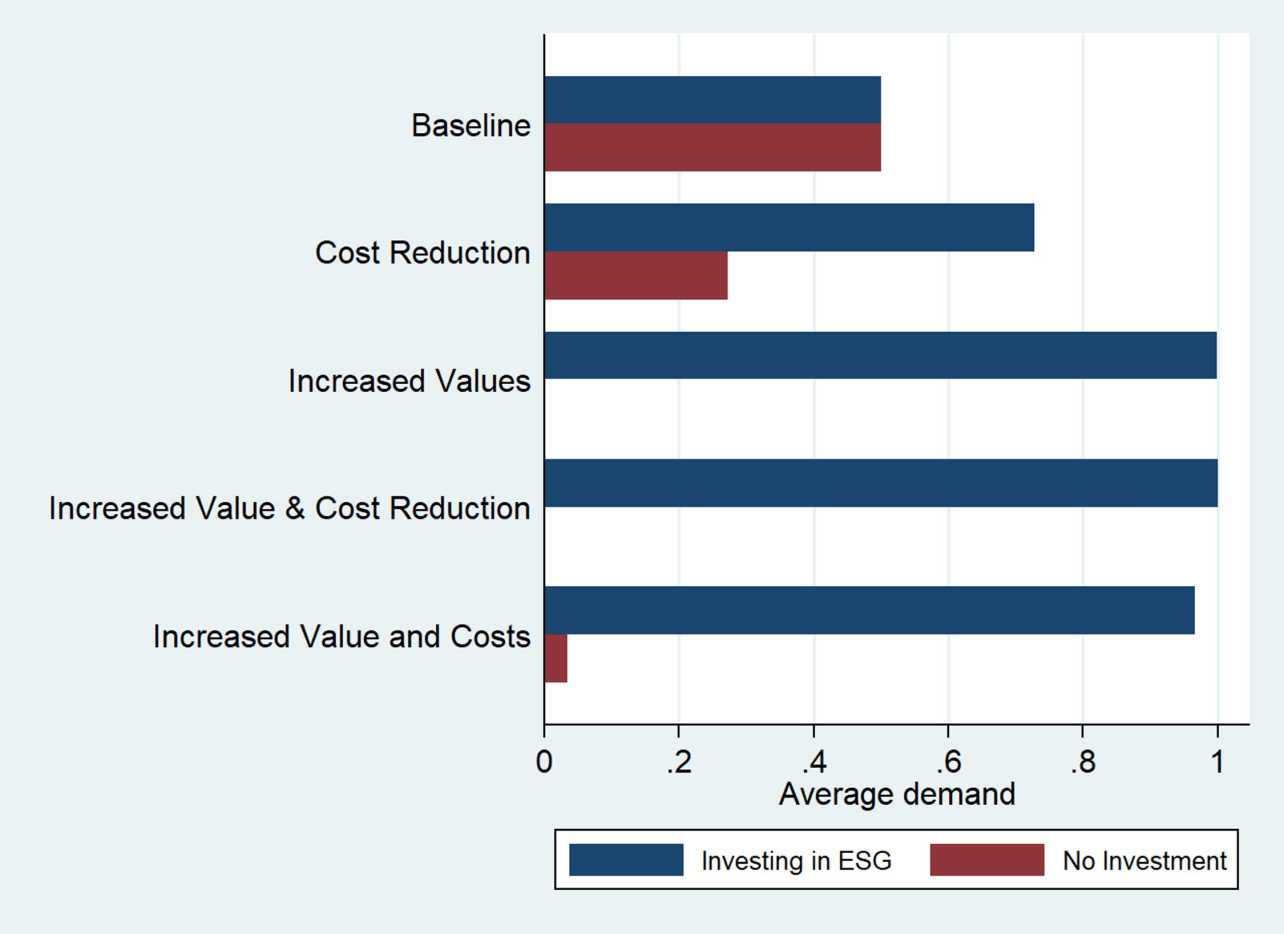
The average demand for each firm under different scenarios when one firm invests in ESG (simultaneous move game).

For example, as marginal costs of production increase, the larger the incentive to invest and the more significant the asymmetry that ESG generates in the market. Fig 8 shows the average demand of each competitor for all the scenarios presented earlier. In all cases, the investing firm either dominates the market or drives out the competitor.

Interestingly, when ESG increases the product value, the firm that invests achieves complete dominance. It sets prices that extract all surplus from consumers and expels the other firm from the market. The main takeaway is that when only one firm invests, the market is likely to converge to a monopoly or to a situation where a leader and a follower coexist. Therefore, when competitors do not invest in ESG, a firm has a strong incentive to invest in ESG, which will help the firm integrate purpose and profit.

## Discussion & conclusion

We discuss theoretical and managerial implications, identify opportunities for future research, and conclude.

### Theoretical and managerial implications

As business interest in ESG increases, firms seek to understand how they can do well by doing good. Because the empirical research finds mixed results on this topic, we need modeling research that elucidates the mechanisms that link ESG investment and firm performance. Our research makes several theoretical and practical contributions to that direction.

We build a computational model that captures ESG investment that attracts consumers. This is a novel concept in the ESG literature, and our work is the first to explore its implications.

This approach offers an integrative framework that allows for studying various ESG effects and their interactions. In our computational model, firms invest in ESG to attract consumers. In addition to that main direct ESG effect, other effects may be present, for example, higher product value, lower production costs, or a combination. Those effects interact with the market structure to determine the competitive outcome, which is a second-order effect of ESG investment.

Our computational scenarios explain the competitive implications of ESG, showing a rationale for commonly observed ESG strategies of firms. Our results show that ESG investment is a pro-competitive force that lowers prices and profits and increases the total value created. It is in firms’ interest to invest in ESG to attract consumers, increase the product value or reduce production costs. However, the profitability of such actions quickly disappears when competitors follow, eroding any first-mover advantage. Under certain conditions, it may lead to an extreme case in which firms compete fiercely, eroding all profits, which highlights the pro-competitive nature of ESG. Although this result seems to discourage ESG investment, if only one firm invests in ESG, it gets ahead of its competitor, who may exit the market. We test different effects and show that they reinforce each other when combined. In other words, competitiveness intensifies when ESG reduces costs and increases willingness to pay, but so does the winner-take-all dynamic when only one firm invests.

However, managers can find ways to mitigate this pro-competitive effect and maintain investment incentives. If firms can pre-announce their ESG projects (investment), prices are higher, and profits do not dissipate completely. Therefore, firms may use strategic disclosure of information to avoid excessive investments and soften competition. In other words, firms have the incentive to signal their ESG investment. This situation opens a new question about how firms may use their marketing campaigns to signal their ESG investment to competitors. While informative campaigns are necessary to raise awareness about ESG practices, or new products, firms can use those campaigns to highlight to what extent they are willing to invest, thus softening competition and reducing the potential increases in welfare that ESG may create.

In contrast, a winner-take-all outcome is likely when only one firm invests in ESG and does not communicate its investment. This finding supports the current industry belief that companies delivering on ESG commitments tend to outperform their competitors [58]. If a company does not act quickly enough, there is a real threat of being left behind in the competition. Therefore, the firm that does not invest in ESG has a strong incentive to start investing.

Managers should be aware that the competitive context (market structure) is a critical determinant of the value that a firm will extract from ESG investments. Particular ESG investments might create significant value for a firm in a monopoly setting, but this value may disappear in a competitive setting. We show that, individually, firms benefit from investing in ESG. However, if all firms do the same, they start competing fiercer. Therefore, managers have to address the trade-offs between purpose and profit. In this regard, a critical message from our results that supports current practices is that companies should communicate ESG investment. These communications help send signals to competitors that moderate competition while promoting ESG investment without eroding profits.

### Limitations and future research

A primary insight of our research is that the competitive setting and the type of ESG investment matter when studying the strategic effects of ESG. That insight suggests opportunities for future research. Future research could relax some model assumptions and consider more competitive settings, such as markets with quality differentiation, network effects, or other consumer behavioral rules and types of ESG investments. Our research focuses on ESG implications for the firms, but future work could also focus on broader social or economic implications. Moreover, this research provides some initial observations into the enabling role of digital technologies in the context of ESG, but more studies are needed [59, 60].

## Conclusions

Τhis study builds a computational model of firms investing in ESG in a competitive setting. The model captures a novel effect of ESG investment: firms invest in ESG to attract consumers. We show that this effect, along with other direct effects, such as increased product value, interacts with the market structure to determine the competitive outcomes of ESG investment.

Our computational experiments show that ESG tends to intensify competition, increasing total and consumer value, but it can also drive winner-takes-all dynamics in asymmetric settings. Our research contributes to the emerging ESG literature and provides new lessons for managers. Overall, our research clarifies the strategic implications of ESG investment and the conditions under which firms can integrate purpose and profit. Lastly, our research shows that computational modeling is a valuable approach to understanding ESG and its impact on competition and industry dynamics.

## Supporting information

S1 File (ZIP)
