# Supplementary material for: A computational model of the competitive effects of ESG
Source: PLoS One. 2023 Jul 21;18(7):e0284237. doi: 10.1371/journal.pone.0284237 (PMC10361511; doi:10.1371/journal.pone.0284237)
Supplement: S1 File — (ZIP) [file pone.0284237.s001.zip › Supporting Information files/S1_Appendix.docx]

# Appendix: Sensitivity analysis of the strength of attracting consumers

All our scenarios have a common effect, ESG attracts consumers. We assumed that the ESG investment unit could change user perception by 3%. To test the robustness of previous insights, we conduct a sensitivity analysis in which we consider that ESG investment could also modify the user perceptions by 6%, 9% and 12%. Intuitively, attracting consumers in some markets may be easier than in others, so it is crucial to see how this affects our key findings.

In general, we find that our prior intuitions are robust. For example, when ESG investment reduces marginal costs, firms invest to the point where their marginal costs are zero. The investment follows the same trend but is more pronounced if we consider that ESG modifies perceptions by more than 3% at each step (Fig 9).


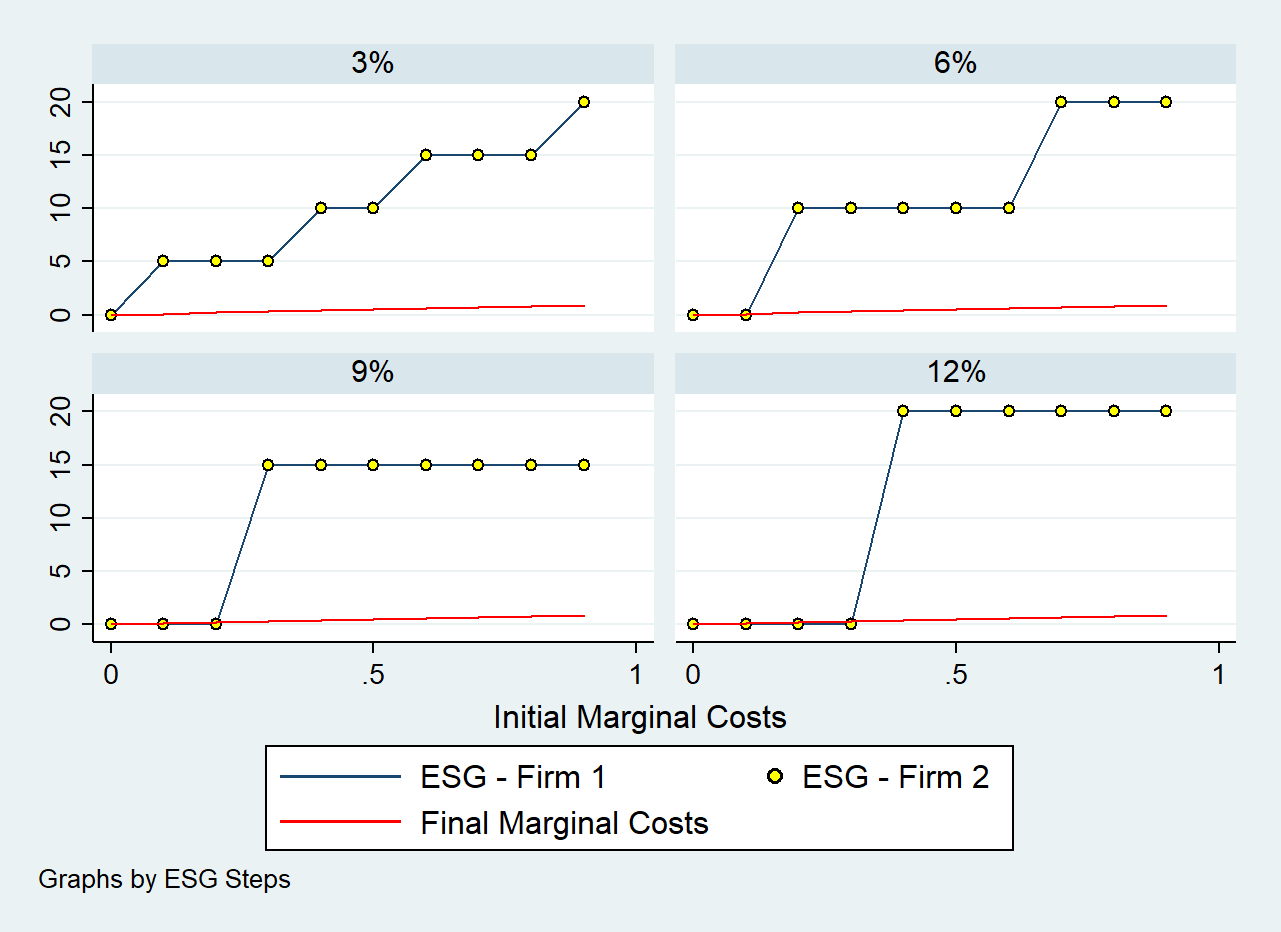


**Fig 9. Sensitivity analysis on ESG investment that reduces costs. Cases by influence of ESG on consumers' perception.**

When ESG increases product value, our previous results still hold but with nuances. When mismatch cost is low, one firm can keep the whole market. However, as we increase differentiation, each firm has an incentive to invest in ESG and keep the competitor out. Our results with ESG steps higher than 3% show a more extreme case than the one presented before –one firm captures the entire market from the beginning. Intuitively, when ESG steps are higher, it is easier for companies to expel the competitor. It implies that as we increase the effect of ESG investments, corner solutions become focal equilibria similar to what would happen if we had strong network effects in digital markets. Fig 10 (upper left, bottom graph) shows that the equilibrium prices are stable only when ESG steps are small. In the rest of the cases, prices always increase, highlighting a corner solution with only one firm.


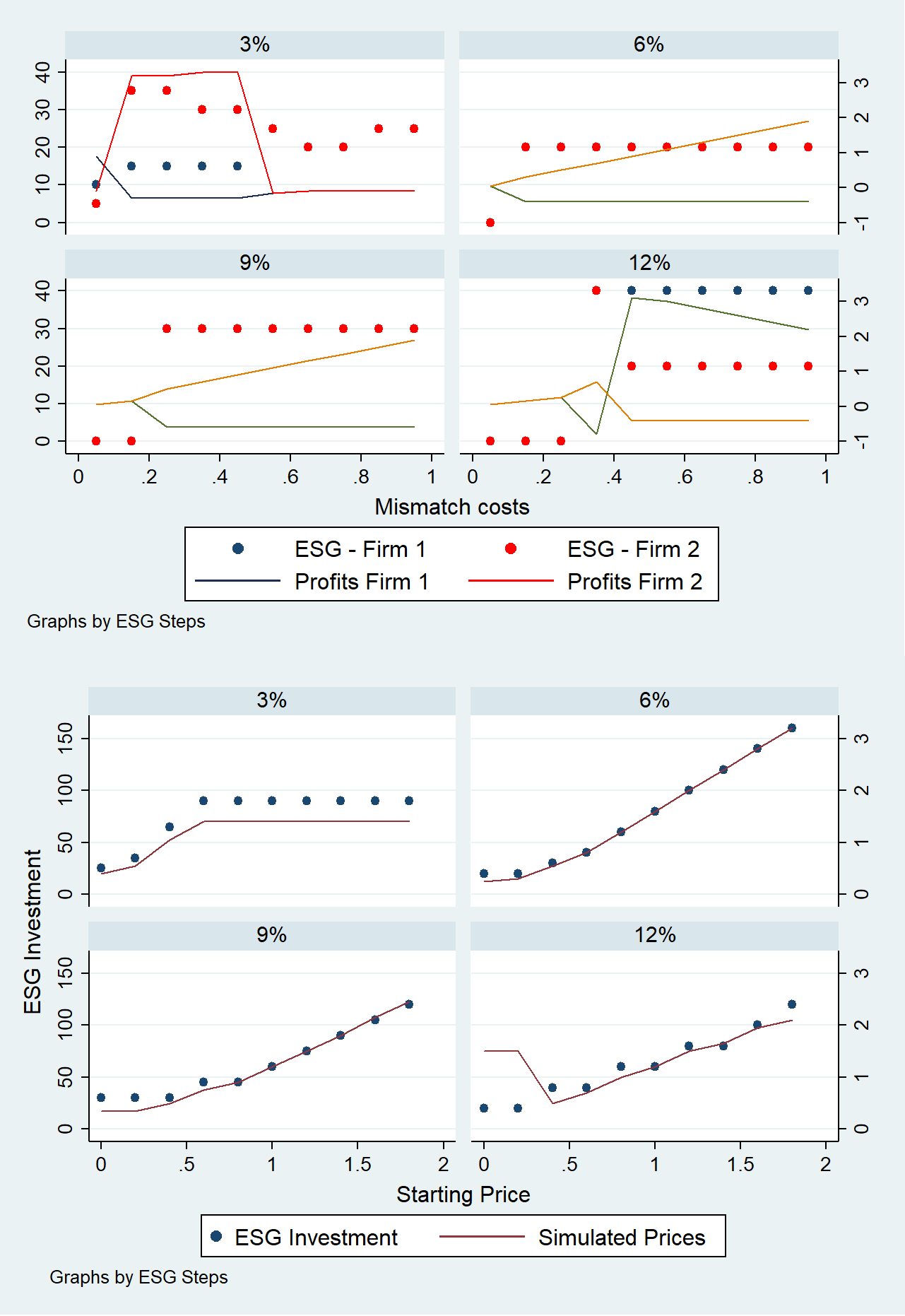


**Fig 10. Sensitivity analysis on ESG investment that increases value. Cases by influence of ESG on consumers' perception.**

When ESG reduces costs and increases product value, we observe a nonlinear impact on prices, no matter which ESG step we use (Fig 11). Nonetheless, there are some differences. In all cases, there is an incentive to invest in ESG. But the higher the step, the lower the investment. This is just a reflection of the discrete nature of the model.


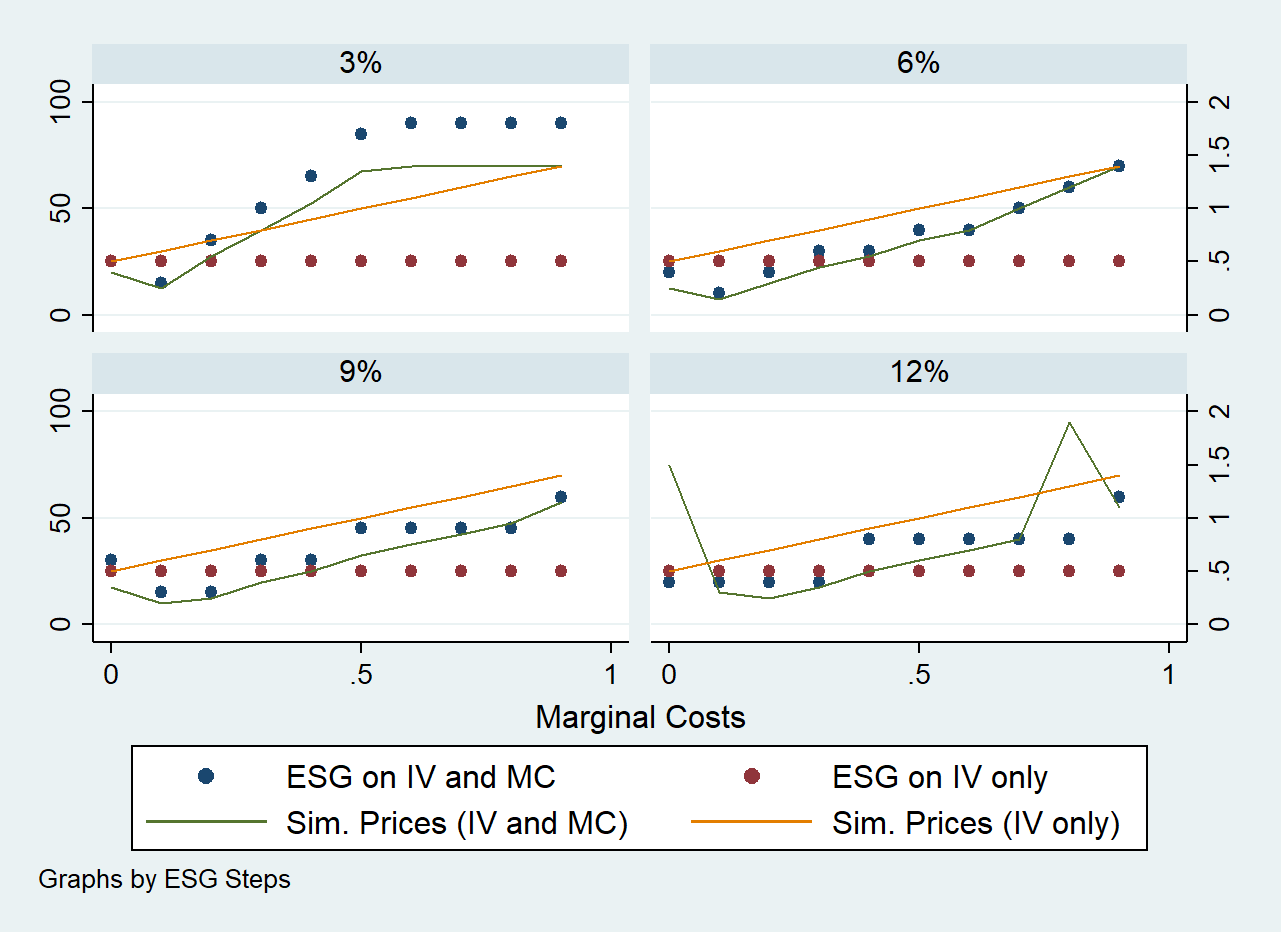


**Fig 11. Sensitivity analysis on ESG investment that reduces costs and increases product value. Cases by influence of ESG on consumers' perception.**

Our previous insights still hold if we assume that producing those products is more expensive (Fig 12). When marginal costs are low, firms invest in ESG because they can profit from the increased intrinsic product value, but both firms dissipate profits. As we previously highlighted, these results suggest that firms with low marginal costs may be more inclined to invest in ESG, even if it implies high costs. Therefore, we can conclude that ESG may not be a one-size-fits-all solution for all companies, and significant differences likely appear depending on the cost structures of each industry.


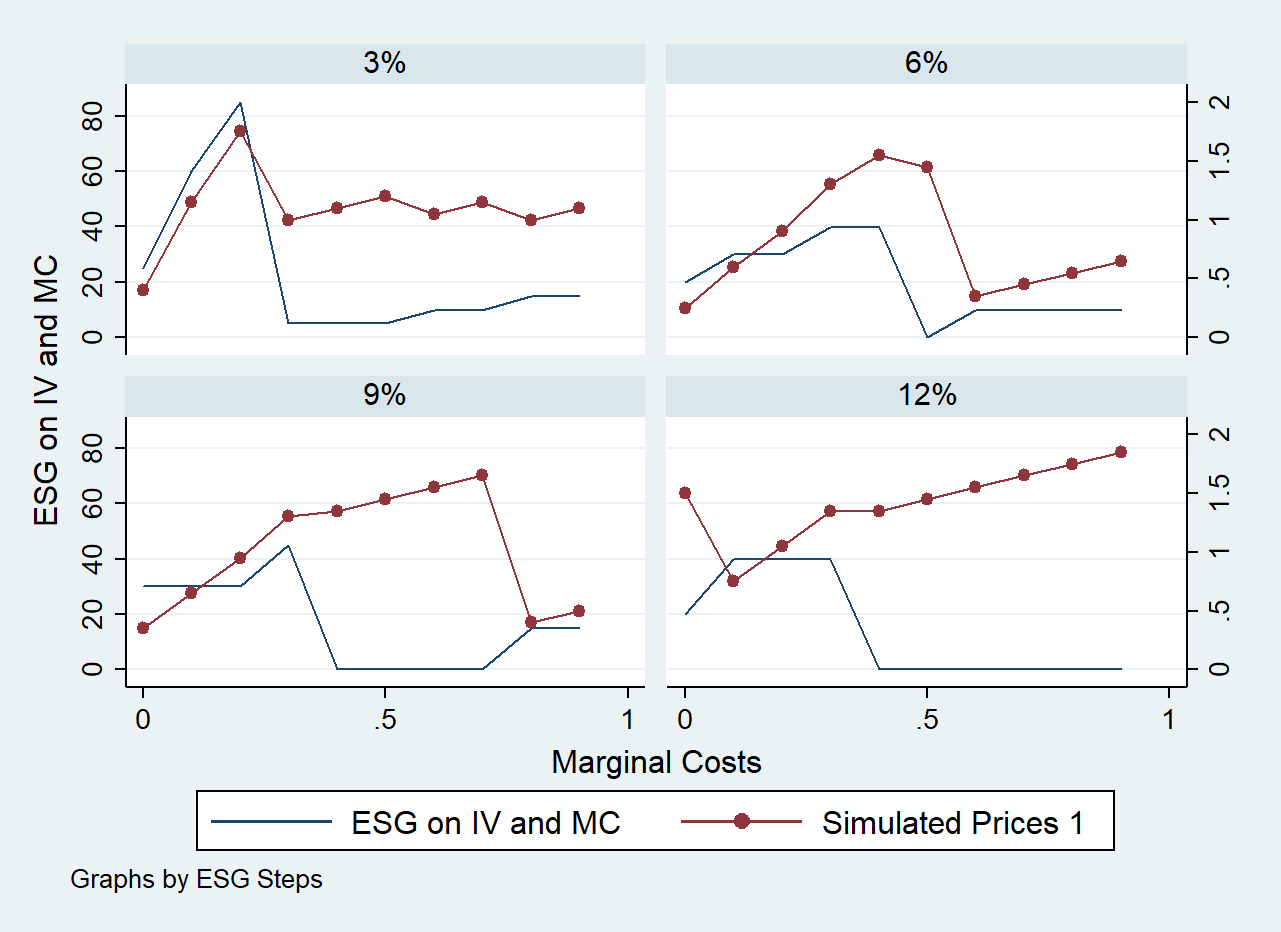


**Fig 12. Sensitivity analysis on ESG investment that increases costs and product value. Cases by influence of ESG on consumers' perception**
